# Updated long-term survival outcomes for patients with locally advanced gastric cancer having pathological complete response after neoadjuvant therapy at China National Cancer Center, 2004–2023

**DOI:** 10.3389/fonc.2025.1539534

**Published:** 2025-06-04

**Authors:** Chongyuan Sun, Tongbo Wang, Xiaojie Zhang, Lulu Zhao, Penghui Niu, Wanqing Wang, Xiaoyi Luan, Xue Han, Yingtai Chen, Dongbing Zhao

**Affiliations:** Department of Pancreatic and Gastric Surgery, National Cancer Center/National Clinical Research Center for Cancer/Cancer Hospital, Chinese Academy of Medical Sciences and Peking Union Medical College, Beijing, China

**Keywords:** gastric cancer, neoadjuvant therapy, pathological complete response (pCR), prognostic factor, immunotherapy

## Abstract

**Background:**

Neoadjuvant chemotherapy increases the probability of achieving negative margins and may even lead to pathological complete response (pCR) in locally advanced gastric cancer (LAGC). The incorporation of neoadjuvant immunotherapy is promising in further enhancing the pCR rate. However, long-term survival outcomes and factors affecting the prognosis of pCR patients have not been fully elucidated.

**Patients and methods:**

We conducted a retrospective analysis of all patients who achieved pCR between January 2004 and June 2023. Cox regression models were used to identify clinicopathological predictors of overall survival (OS) and disease-free survival (DFS). Survival curves were plotted using the Kaplan–Meier method and compared using the log-rank test.

**Results:**

After screening, 112 patients were included in the study, with a median follow-up time of 42 (range: 2-117) months and a pCR rate of 7.4%. The 3- and 5-year OS rates were 90.2% and 83.3%, respectively, while the 3- and 5-year DFS rates were 86.8% and 82.0%, respectively. Within the multivariate Cox model, neoadjuvant chemotherapy was a prognostic factor for improved OS and DFS. There was no statistically significant disparity in OS and DFS between patients who received postoperative adjuvant therapy and those who did not. Moreover, the combination of neoadjuvant immunotherapy with chemotherapy, as compared to neoadjuvant chemotherapy alone, substantially increased the pCR rate (p <0.001).

**Conclusions:**

Patients with LAGC who achieved pCR demonstrated favorable long-term survival outcomes, with no additional survival benefits conferred by adjuvant therapy. Although neoadjuvant immunotherapy increased the pCR rate, its impact on the prognosis of pCR patients requires further investigation.

## Introduction

Gastric cancer (GC) is the fifth most prevalent malignancy worldwide (1), with a generally poor prognosis (2). Following the landmark MAGIC trial (3–5), neoadjuvant therapy (NAT) has been recommended by numerous clinical practice guidelines (6–8), and has gradually becomes the standard treatment for patients with locally advanced gastric cancer (LAGC) (9). This transformation can be attributed to its multifaceted advantages, including a higher likelihood of achieving negative margins, tumor downstaging and improvements in both disease-free survival (DFS) and overall survival (OS) (10, 11).

Pathological complete response (pCR), defined as the absence of any viable tumor cells in the affected tissue (12), has been associated with improved survival in various cancers, including breast (13), rectal (14), esophageal (15), and bladder cancer (16). However, the prognostic significance of pCR in LAGC remains inadequately elucidated. In 2019, Wang reported on a cohort of 39 patients achieving pCR following NAT at the China National Cancer Center, revealing 3- and 5-year OS rates of 88.9% and 88.9%, respectively (17). While recent publications have summarized the characteristics and survival outcomes of pCR in LAGC patients, these studies were limited by small sample sizes and the inclusion of patients with lymph node metastasis (ypN+), which may affect the generalizability of their findings (18–20). Moreover, combined immunotherapy and chemotherapy showed prolonged OS and progression-free survival (PFS) compared to chemotherapy alone in patients with PD-L1 CPS ≥5 in CheckMate 649 (21) and ORIENT-169 (22). Ongoing investigations of neoadjuvant immunotherapy in LAGC hold the potential to further increase the pCR rate (23, 24).

This study aims to update our previous findings by including a larger cohort of 112 patients with pCR status between January 2004 and June 2023, providing a more robust analysis of long-term outcomes. Furthermore, we explored the potential benefits of postoperative adjuvant therapy and the early efficacy of neoadjuvant immunotherapy.

## Methods

### Patients and study design

A retrospective review was conducted on patients diagnosed with LAGC who underwent NAT and achieved pCR at our center between January 2004 and June 2023. The inclusion criteria were as follows: (1) histological confirmation of gastric adenocarcinoma via gastrointestinal endoscopy before treatment; (2) locally advanced disease (cT3–4 and/or N+) according to the 8th edition of the American Joint Committee on Cancer (AJCC) TNM staging system (3) administration of systemic chemotherapy or concurrent chemoradiotherapy prior to surgery; and (4) histological grade of tumor regression after surgical resection classified as complete regression based on the Mandard tumor regression grade (TRG). Exclusion criteria included patients with non-adenocarcinomatous histology and distant metastasis (M1). All study procedures were approved by the Institutional Review Board at the China National Cancer Center.

The primary endpoint of this study was to assess the 5-year OS and DFS of patients with LAGC who achieved pCR. Secondary endpoints included identifying independent prognostic factors in pCR patients. Additionally, pCR rates in patients who received neoadjuvant chemotherapy alone followed by surgery were compared with those undergoing combined neoadjuvant immunotherapy and chemotherapy followed by surgery.

### Neoadjuvant therapy

All patients in the study received NAT, which consisted of either neoadjuvant chemotherapy (nCT) or neoadjuvant chemoradiotherapy (nCRT). A multidisciplinary team, including gastrointestinal surgeons, radiologists, medical oncologists, and radiation oncologists, reviewed each case to determine the optimal therapeutic strategy.

Patients receiving preoperative chemotherapy were administered various regimens, including FOLFOX (oxaliplatin/5-fluorouracil [5-Fu]/folinic acid), FLOT (oxaliplatin/5-Fu/folinic acid/docetaxel), SOX (oxaliplatin/S-1 [an oral 5-Fu prodrug]), XELOX (oxaliplatin/capecitabine), paclitaxel/oxaliplatin, DOS (docetaxel/oxaliplatin/S-1), and others. The dosage and duration of treatment were adjusted as needed in cases of intolerance or severe adverse events. Typically, 3 or 4 cycles of nCT were recommended. In some instances, patients also received immunotherapy during nCT, specifically programmed cell death protein-1 (PD-1) inhibitors. Patients undergoing neoadjuvant concurrent chemoradiotherapy received a total dose of 45Gy delivered in 25 fractions of 1.8 Gy over 5 weeks, along with concurrent administration of S-1 at a dose of 80 mg/m^2^.

### Surgical treatment and histological examination

Resectable patients underwent radical gastrectomy with D2 lymphadenectomy approximately 4–6 weeks after completing NAT. Histologic regression was evaluated using the Mandard TRG (25) as follows: TRG 1 (complete regression) showed absence of residual cancer and fibrosis extending through the different layers of the gastric wall; TRG 2 was characterized by the presence of rare residual cancer cells scattered through the fibrosis; TRG 3 was characterized by an increase in the number of residual cancer cells, but fibrosis still predominated; TRG 4 showed residual cancer outgrowing fibrosis; and TRG 5 was characterized by absence of regressive changes. pCR was defined as TRG 1 at the primary tumor site (T0) with no regional lymph node metastasis (N0).

### Postoperative adjuvant therapy and outcome measurement

Adjuvant chemotherapy was recommended for all patients enrolled in the study. However, 33 patients declined adjuvant therapy due to various personal reasons, and 10 patients received only postoperative immunotherapy.

OS was defined as the time from the initiation of NAT to death, while DFS was defined as the time from the initiation of NAT to either tumor recurrence or death, whichever occurred first. Patient survival status was obtained through telephone interviews and outpatient records, with the follow-up period ending in June 2023.

### Statistics

Continuous variables are presented as medians with ranges, and categorical variables as counts with percentages. Comparisons of categorical variables between groups were performed using chi-square tests or Fisher’s exact tests, as appropriate. OS and DFS rates were calculated using the Kaplan–Meier method. Univariate and multivariate Cox regression analysis was performed to identify factors affecting patient prognosis. Variables with a p-value of less than 0.05 in the univariate analyses were further evaluated in the multivariate analysis. All statistical tests were two-sided, and a p-value of less than 0.05 was deemed statistically significant. Analyses were performed using IBM SPSS Statistics 26.0 (IBM Corp., Armonk NY, USA), and graphs were generated using R software (version 4.2.2, R Foundation for Statistical Computing, Vienna, Austria).

## Results

### Patient characteristics

Overall, 1517 consecutive patients with gastric cancer who received NAT at our center underwent screening. Of these, 124 patients achieved ypT0N0 status, and 12 were excluded due to liver metastasis or distant lymph node metastasis(M1). Consequently, 112 pCR patients (112/1517; 7.4%) were deemed eligible for the study. The demographic and clinical characteristics of these patients are summarized in **Table 1**. The median age at diagnosis was 60 years (range 22–76), with 87 out of the 112 patients being male (77.7%). Nearly half of the patients (51/112; 45.5%) had carcinoma in the lower stomach, followed by tumors in the upper stomach (41/112; 36.6%). The majority of patients (79/112; 70.5%) were diagnosed with poorly differentiated adenocarcinoma, while moderately differentiated tumors were detected in 15 patients (13.4%). The differentiation of endoscopic biopsy was not graded in 18 patients. No significant differences were observed between the nCT alone group and the nCT combined with immunotherapy group (all p >0.05).

**Table 1:** Demographic and tumor characteristics of the patients.

Variable	No. (%)	nCT alone (n=56)	nCTI (n=39)	p value
Age (years)				0.166
≥65	40 (35.7)	22 (39.3)	10 (25.6)	
<65	72 (64.3)	34 (60.7)	29 (74.4)	
Sex				0.154
Male	87 (77.7)	47 (83.9)	28 (71.8)	
Female	25 (22.3)	9 (16.1)	11 (28.2)	
Tumer location				0.641
Upper	41 (36.6)	24 (42.9)	13 (33.3)	
Lower	51 (45.5)	25 (44.6)	20 (51.3)	
Diffuse	20 (17.9)	7 (12.5)	6 (15.4)	
Histologic grade				0.315
Moderately differentiated	15 (13.4)	6 (10.7)	8 (20.5)	
Poorly differentiated	79 (70.5)	38 (67.9)	28 (71.8)	
Unknown	18 (16.1)	12 (21.4)	3 (7.7)	
Signet ring cell morphology				0.106
Yes	15 (13.4)	5 (8.9)	8 (20.5)	
No	97 (86.6)	51 (91.1)	31 (79.5)	
cT stage				0.653^a^
2	5 (4.5)	3 (5.4)	1 (2.6)	
3	36 (32.1)	19 (33.9)	11 (28.2)	
4	71 (63.4)	34 (60.7)	27 (69.2)	
cN stage				0.077^a^
0	13 (11.6)	9 (16.1)	3 (7.7)	
1	33 (29.5)	17 (30.4)	8 (20.5)	
2	49 (43.7)	25 (44.6)	17 (43.6)	
3	17 (15.2)	5 (8.9)	11 (28.2)	
cTNM stage				0.059
II	32 (28.5)	20 (35.7)	7 (17.9)	
III	80 (71.5)	36 (64.3)	32 (82.1)	

nCT alone neoadjuvant chemotherapy alone, nCTI neoadjuvant chemotherapy combined with immunotherapy.

a Fisher's exact tests.

### Perioperative therapy and surgical treatment

**Figure 1:**
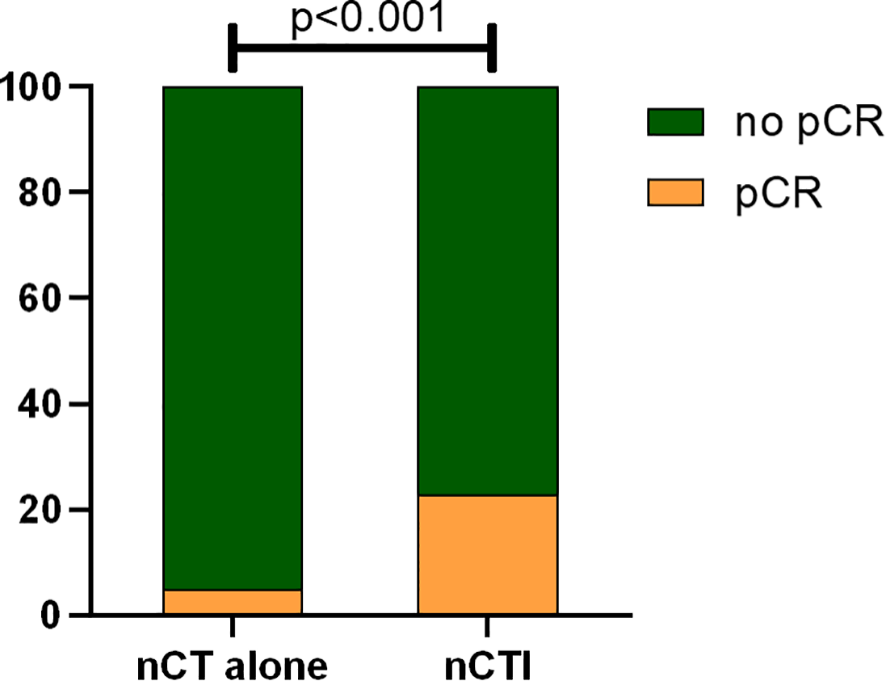
Comparison of pCR rates between neoadjuvant chemotherapy alone and neoadjuvant chemotherapy combined with immunotherapy (pCR pathological complete regression, nCT alone neoadjuvant chemotherapy alone, nCTI neoadjuvant chemotherapy combined with immunotherapy).

**Table 2** summarizes the treatment regimens for all 112 patients. The nCT group included 95 patients treated with various chemotherapeutic regimens, including SOX (n = 37), XELOX (n = 11), paclitaxel/oxaliplatin (n = 6), DOS (n = 24), and others. Of the 39 patients who received concurrent immunotherapy, 12 were treated with sintilimab, 8 with camrelizumab, 6 with pembrolizumab, 5 with toripalimab, 3 with tislelizumab, 2 with nivolumab, 2 with penpulimab, and 1 with durvalumab. The median number of nCT cycles administered was 4 (range: 2–11). Compared to nCT alone, the combination of neoadjuvant immunotherapy and chemotherapy led to a remarkable increase in the pCR rate, elevating it from 4.7%(56/1185) to 22.7% (39/172) (p <0.001; **Figure 1**).

**Table 2:** Perioperative treatment patterns and surgical characteristics.

Characteristic	No.
NAT pattern	
Chemotherapy	95
FOLFOX	1
XELOX	11
FLOT	2
DOC	5
DS	1
DCS	1
DOF	1
Paclitaxel/capecitabine	1
SOX	37
Paclitaxel/oxaliplatin	6
DOS	24
Unknown^a^	5
Concurrent chemoradiotherapy	17
Cycles of nCT,median[range]	4[2-11]
Gastrectomy type	
Proximal gastrectomy	28
Distal gastrectomy	49
Total gastrectomy	35
No. of LNs examined	
<16	5
≥16	107
Adjuvant therapy	
No	33
Platinum + fluoropyrimidine	34
Taxane based	13
S-1/capecitabine	13
Immunotherapy	10
Unknown^a^	9

NAT, neoadjuvant therapy; FOLFOX, oxaliplatin/5-Fu/folinic acid; XELOX, oxaliplatin/capecitabine; FLOT, oxaliplatin/5-Fu/folinic acid/ docetaxel; DOC, docetaxel/oxaliplatin/capecitabine; DS, docetaxel/S-1; DCS, docetaxel/cisplatin/S-1; DOF, docetaxel/oxaliplatin/5-Fu; SOX, S-1/oxaliplatin; DOS, docetaxel/oxaliplatin/S-1; LNs, lymph nodes.

a Chemotherapy regimen unknown.

All 112 patients underwent radical gastrectomy with D2 lymphadenectomy, including proximal gastrectomy in 28 patients, distal gastrectomy in 49, and total gastrectomy in 35. Notably, all surgical procedures achieved R0 resection, and the median number of lymph nodes detected was 32 (range: 7–101). Regarding postoperative adjuvant therapy, 79 individuals (70.5%) received adjuvant therapy, consisting of 34 patients with the regimen of platinum/fluoropyrimidine, 13 with taxane-based regimens, 13 with S-1/capecitabine and 10 with single-agent immunotherapy. Thirty-three patients (29.5%) did not receive adjuvant therapy after surgical resection.

### Long−term survival outcome

**Figure 2:**
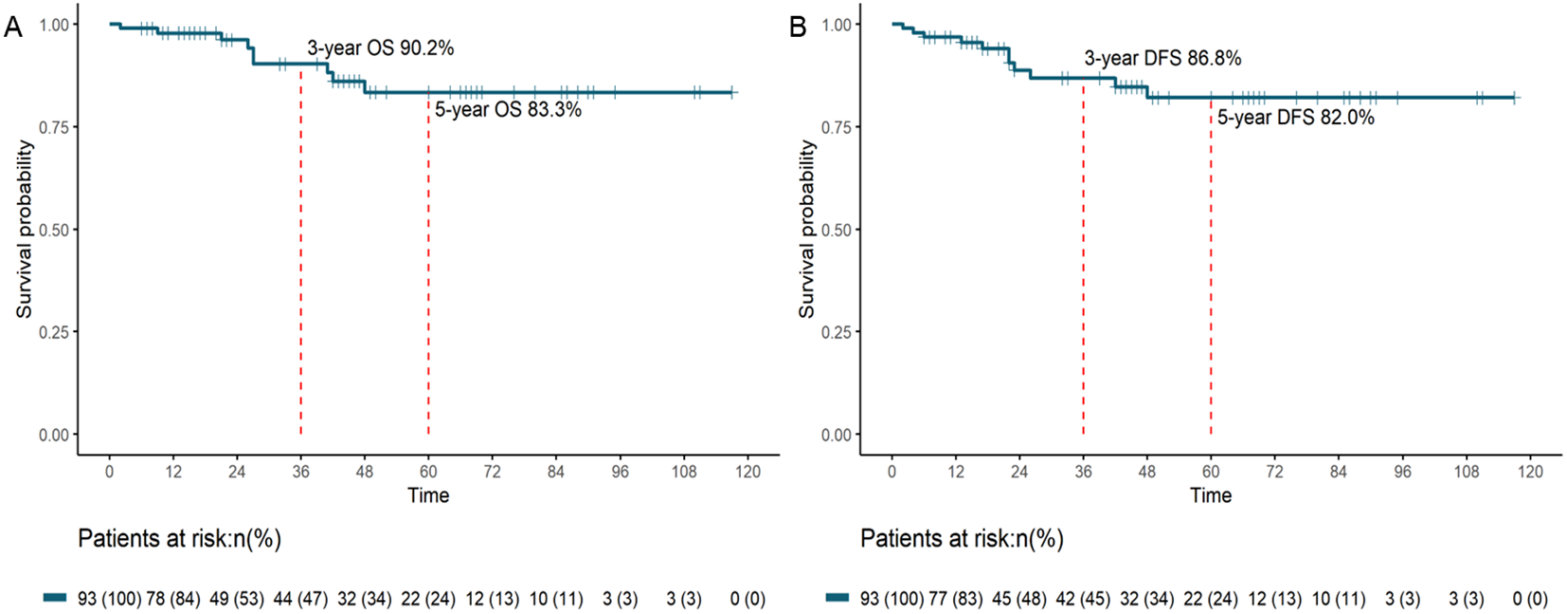
OS **(A)** and DFS **(B)** survival curves for patients with pCR after neoadjuvant therapy and resection (OS, overall survival; DFS, disease-free survival; pCR, pathological complete response).

With a median follow-up duration of 42 (range: 2–117) months, both median OS and DFS were not reached. The 3-year OS rate was 90.2%, and the 5-year OS rate was 83.3% (**Figure 2A**). The 3-year DFS rate was 86.8%, and the 5-year DFS rate was 82.0% (**Figure 2B**).

Recurrence was observed in 8 patients, among which 7 patients received adjuvant chemotherapy and 1 of them did not. Recurrence sites included abdominal lymph nodes in 2 cases, pleural involvement in 4 cases, brain metastases in 2 cases, bone metastases in 1 case, and other locations (**Table 3**). The median time to recurrence was 22 months (range: 4–26).

**Table 3:** Characteristics of patients with recurrence.

Case No.	NAT	AT	Recurrence site	DFS(month)	Status at last follow-up	Time from recurrence to death or last follow-up (months)
1	nCRT	No	Pleura	22	DOD	4
2	nCRT	Yes	Pleura, colon	23	DOD	18
3	nCT	Yes	Pleura, lung	26	DOD	1
4	nCRT	Yes	Pleura	13	DOD	8
5	nCT	Yes	brain	6	DOD	3
6	nCRT	Yes	bone, supraclavicular lymph nodes	4	DOD	23
7	nCT	Yes	Residual stomach, abdominal lymph nodes, brain	22	AWD	20
8	nCT	Yes	Abdominal lymph nodes	17	AWD	1

NAT, neoadjuvant therapy; AT, adjuvant therapy; nCRT, neoadjuvant chemoradiotherapy; nCT, neoadjuvant chemotherapy; DOD, dead of disease; AWD, alive with disease.

### Prognostic factors in patient survival

**Figure 3:**
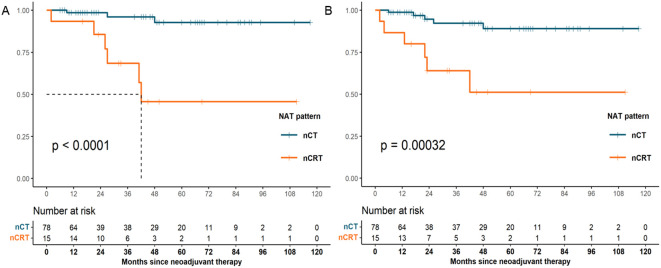
OS **(A)** and DFS **(B)** survival curves for patients with pCR who received nCT and those who received nCRT (OS, overall survival; DFS, disease-free survival; pCR, pathological complete response; nCT, neoadjuvant chemotherapy; nCRT, neoadjuvant chemoradiotherapy).

**Figure 4:**
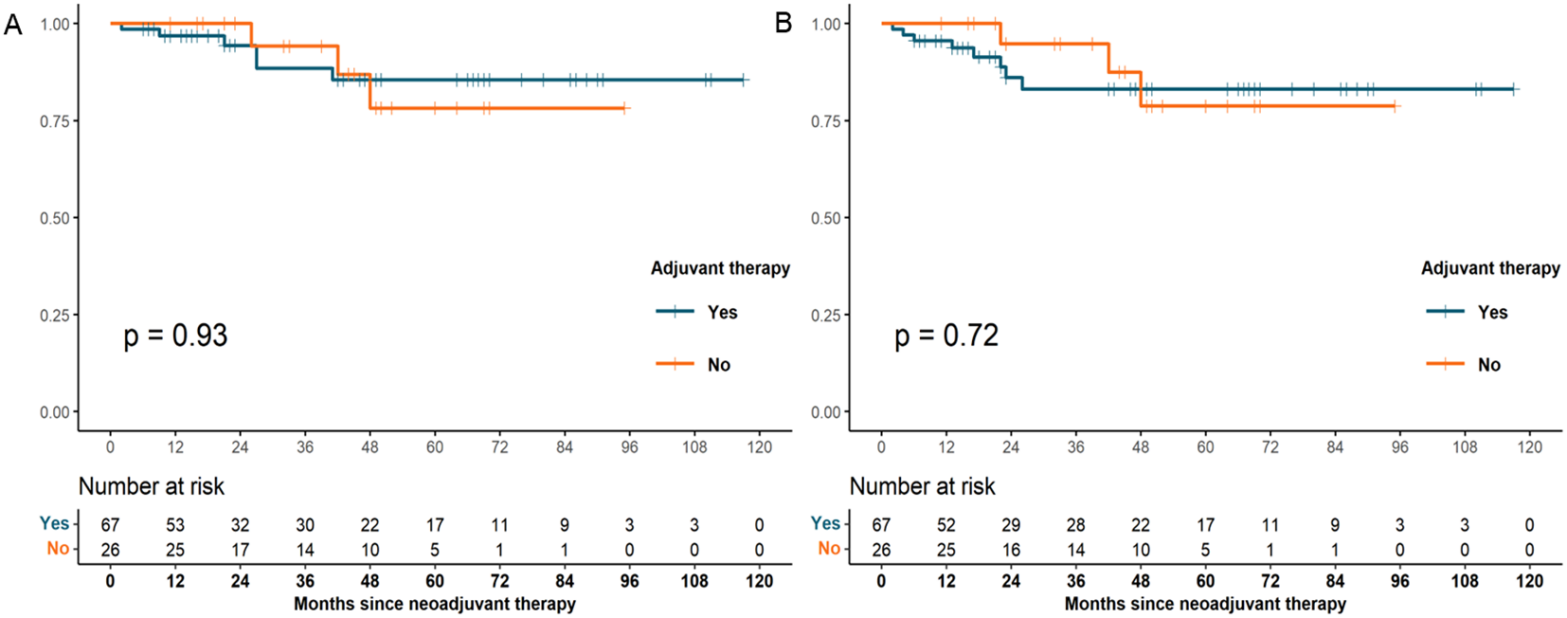
OS **(A)** and DFS **(B)** survival curves for patients with pCR who received adjuvant therapy and those who did not (OS, overall survival; DFS, disease-free survival; pCR, pathological complete response).

Cox regression analysis was performed to assess the association between patients’ demographics, tumor characteristics, and treatment factors with survival outcomes. In univariate analysis, we observed that tumor location (p = 0.02) and NAT pattern (hazard ratio [HR] 10.756, 95% confidence interval [CI] 2.653–43.604; p = 0.001) were related to OS. However, factors including age at diagnosis, sex, histologic grade, clinical T category, clinical N category, type of resection, number of lymph nodes examined, nCT cycles, and adjuvant therapy was not significant associated with OS (p >0.05). In the multivariate Cox model, NAT pattern (nCT vs. nCRT: HR 7.093, 95% CI 1.470–34.229; p = 0.015) was confirmed as significant prognostic factor for OS (**Table 4**). The impact of different NAT patterns and the receipt of adjuvant therapy on OS was illustrated in **Figure 3A** and **Figure 4A**, respectively.

**Table 4:** Univariate and multivariate Cox regression analyses of overall survival in pCR patients.

Variable	Univariable analysis		Multivariable analysis	
	HR (95% CI)	p value	HR (95% CI)	p value
Age at diagnosis (≥65 vs. <65 years)	1.492 (0.400-5.561)	0.551		
Sex (female vs. male)	0.525 (0.066-4.202)	0.544		
Tumor location (ref. Upper)		**0.020**		0.373
Lower	0.821 (0.115-5.837)	0.844	0.715 (0.099-5.158)	0.740
Diffuse	5.915 (1.145-30.541)	0.034	2.352 (0.388-14.263)	0.352
Histologic grade (Moderatel differentiated vs. poorly differentiated)	2.489 (0.296-20.909)	0.401		
Signet ring cells (present vs. absent)	1.372 (0.170-11.097)	0.767		
Clinical T category (ref. T4)		1.000		
T2	<0.001^a^	0.987		
T3	1.014 (0.253-4.054)	0.985		
Clinical N category (ref. N2)		0.936		
N0	0.870 (0.097-7.823)	0.901		
N1	1.490 (0.371-5.974)	0.574		
N3	<0.001^a^	0.986		
Type of resection (ref. Distal gastrectomy)		0.139		
Proximal gastrectomy	1.258 (0.176-8.970)	0.819		
Total gastrectomy	4.225 (0.818-21.826)	0.085		
No.of lymph nodes examined (≥16 vs.<16)	3.309 (0.676-16.207)	0.140		
NAT pattern (nCT vs. nCRT)	10.756 (2.653-43.604)	**0.001**	7.093 (1.470-34.229)	**0.015**
Neoadjuvant chemotherapy cycles (≥4 vs. <4)	0.241 (0.030-1.959)	0.183		
Adjuvant therapy (yes vs. no)	0.940 (0.235-3.763)	0.930		

pCR, pathological complete regression; NAT, neoadjuvant therapy; nCRT, neoadjuvant chemoradiotherapy; nCT, neoadjuvant chemotherapy.

a HR<0.001 and 95% CI not available.

Bold values indicate p < 0.05.

A similar analysis was performed for DFS. NAT pattern was also confirmed as the prognostic factor in both univariate (HR 6.671, 95% CI 2.022-22.016; p = 0.002) and multivariate (HR 4.818, 95% CI 1.267–18.315; p = 0.021) analyses (**Table 5**). The effect of different NAT patterns and the receipt of adjuvant therapy on DFS was presented in **Figure 3B** and **Figure 4B**, respectively.

**Table 5:** Univariate and multivariate Cox regression analyses of disease-free survival in pCR patients.

Variable	Univariable analysis		Multivariable analysis	
	HR (95% CI)	p value	HR (95% CI)	p value
Age at diagnosis (≥65 vs. <65 years)	2.222 (0.678-7.287)	0.187		
Sex (female vs. male)	0.385 (0.0491-3.005)	0.362		
Tumor location (ref. Upper)		**0.025**		
Lower	0.407 (0.074-2.222)	0.299		
Diffuse	3.386 (0.909-12.618)	0.069		
Histologic grade (Moderatel differentiated vs. poorly differentiated)	1.669 (0.203-13.693)	0.633		
Signet ring cells (present vs. absent)	1.074 (0.136-8.454)	0.906		
Clinical T category (ref. T4)		0.668		
T2	<0.001^a^	0.987		
T3	1.723 (0.526-5.648)	0.369		
Clinical N category (ref. N2)		0.891		
N0	1.846 (0.336-10.130)	0.481		
N1	1.519 (0.379-6.085)	0.555		
N3	1.160 (0.129-10.411)	0.895		
Type of resection (ref. Distal gastrectomy)		**0.025**		0.220
Proximal gastrectomy	2.460 (0.450-13.444)	0.299	1.723 (0.403-7.363)	0.463
Total gastrectomy	8.330 (1.614-42.993)	0.011	0.374 (0.068-2.058)	0.258
No.of lymph nodes examined (≥16 vs.<16)	4.826 (0.994-23.426)	0.051		
NAT pattern (nCT vs. nCRT)	6.671 (2.022-22.016)	**0.002**	4.818 (1.267-18.315)	**0.021**
Neoadjuvant chemotherapy cycles (≥4 vs. <4)	1.480 (0.382-5.739)	0.571		
Adjuvant therapy (yes vs. no)	1.279 (0.339-4.832)	0.717		

pCR, pathological complete regression; NAT, neoadjuvant therapy; nCRT, neoadjuvant chemoradiotherapy; nCT, neoadjuvant chemotherapy.

a HR<0.001 and 95% CI not available.

Bold values indicate p < 0.05.

## Discussion

In the present study, we updated and expanded the previously published experience of our institution regarding the long-term outcomes of LAGC patients who achieved pCR after NAT. To our knowledge, this cohort of 112 patients represents the largest single-center study to date focusing on pCR in LAGC. Comparable to our institution’s previously published values, we reported 3- and 5-year OS rates of 90.2% and 83.3%, respectively, along with 3- and 5-year DFS rates of 86.8% and 82.0%, respectively. Moreover, we demonstrated that combining neoadjuvant immunotherapy with chemotherapy significantly improved pCR rate, while the administration of adjuvant therapy did not confer additional survival benefits for pCR patients.

Our study observed favorable survival outcomes among patients with pCR, consistent with findings reported by other researchers. Guo et al. revealed that patients achieving pCR following nCT had 3- and 5-year overall survival rates of 88.8% and 78.6%, respectively, along with 3- and 5-year progression-free survival rates of 86.5% and 75.8%, respectively (20). In a multicenter analysis involving 130 patients with LAGC who received nCT, Lin et al. reported that pCR was an independent prognostic factor for both OS and DFS (26). The survival benefit associated with pCR is theoretically attributed to the simultaneous eradication of the primary tumor, regional lymph node metastases, and potential distant micrometastases through neoadjuvant therapy (27). When pCR is achieved, neoadjuvant therapy may also eliminate occult micrometastatic disease, such as disseminated tumor cells within the peritoneal cavity, thereby exerting a potential curative effect (28). This mechanism may partially account for the long-term survival advantage observed in patients who achieve pCR following NAT.

Previous research has indicated a relatively low pCR rate following nCT, ranging from 4% to 9% (29, 30). Improving the pCR rate remains a critical challenge in clinical practice. In recent years, the emergence of immunotherapy has transformed the standards and principles of cancer treatment, representing the third paradigm shift in oncology after traditional chemotherapy and targeted therapies (31). The Checkmate-649 and ORIENT-16 trials have demonstrated significant clinical benefits and manageable safety profiles when using immune combination therapy as the first-line treatment for advanced gastric cancer (21, 22). The exploration of immune-based regimens in the NAT is currently underway and attracting considerable attention. A single-arm, phase II study evaluating XELOX combined with sintilimab as NAT for potentially resectable gastric or gastroesophageal junction (GEJ) adenocarcinoma showed an R0 resection rate of 97.2%. Among the 36 patients enrolled in the study, 7 (19.4%) achieved a pathological complete response (pCR), with 17 (47.2%) exhibiting a major pathological response (32). Another study found that, in comparison to the SOX or FOLFOX neoadjuvant chemotherapy regimens, the addition of tislelizumab significantly elevated the rate of pCR (3.4% vs. 26.0%; p <0.001) and the rate of R0 resection (89.9% vs. 100%; p = 0.019) in patients with LAGC (33). Li et al. investigated the incorporation of an anti-angiogenic agent (apatinib) into neoadjuvant immunotherapy (carrelizumab) combined with chemotherapy (S-1 ± oxaliplatin), and reported complete and major pathological response rates of 15.8% and 26.3%, respectively (23). Our study yielded similar results, showing that neoadjuvant immunotherapy combined with chemotherapy elevated the pCR rate from 4.7% observed during neoadjuvant chemotherapy to 22.7% (p <0.001). Thus, the integration of neoadjuvant chemotherapy and immunotherapy presents a promising therapeutic approach for patients with gastric cancer.

We also observed an unfavorable prognosis among pCR patients who experienced postoperative recurrence in our clinical practice, with 8 individuals developing recurrence within a median period of less than 2 years. Fields et al. found that despite achieving pCR following NAT, patients still face a significant risk of recurrence and cancer-specific death after resection (34). Therefore, we conducted univariate and multivariate Cox analyses, which revealed that nCT was an independent protective factor for long-term survival, while nCRT was associated with poorer outcomes even with pCR achieved. Previous investigations similarly suggested that nCRT was associated with a higher incidence of pCR compared to nCT, but this improvement did not translate into better prognosis (35). The JCOG1109 NExT study demonstrated that in locally advanced oesophageal squamous cell carcinoma (OSCC), the pCR rate was significantly higher in the NeoCF+RT (cisplatin/fluorouracil + radiotherapy) group compared to the NeoCF (cisplatin/fluorouracil) group (32.5% vs 2.0%), whereas no significant differences were observed in OS (68.3% vs 62.6%, p = 0.12) or PFS (58.5% vs 47.7%) (36). Similarly, the German POET study reported a higher pCR rate in the nCRT group for gastric cancer, but 3-year DFS was comparable between the two groups (37). The potential reason for this discrepancy in outcomes may be attributed to the distinct mechanisms of chemotherapy and chemoradiotherapy. Chemotherapy is a systemic therapy, while chemoradiotherapy offers targeted and localized therapy. Gastric cancer recurrences primarily occur at distant sites, particularly peritoneal implantation metastasis, rather than local recurrence (38). This disparity may explain the improved survival observed after nCT due to its systemic effects. With the advent of the immunotherapy era, it remains unclear whether the increased pCR rates achieved with neoadjuvant immunotherapy will ultimately translate into survival benefits (39). The multicenter phase III KEYNOTE-585 trial demonstrated that perioperative pembrolizumab combined with FOLFOX or FLOT significantly increased the pCR rate compared with FLOT alone (13.4% vs. 2.0%) in patients with resectable locally advanced gastric or gastroesophageal junction cancer, although the improvement in overall survival remained limited (71.8 vs. 55.7 months; HR: 0.86, 95% CI: 0.71–1.06) (40). Similarly, a multicenter study reported a lower early recurrence rate in the neoadjuvant immunochemotherapy group than in the chemotherapy-alone group (29.7% vs. 40.8%, P=0.047), with immunotherapy identified as an independent protective factor (OR: 0.62, 95% CI: 0.41–0.92, P=0.018) (41). However, the limited follow-up duration in this retrospective study warrants further validation of its long-term outcomes.

Another important consideration is whether adjuvant therapy is necessary following pCR. Some data suggested that patients with gastroesophageal cancer who underwent NAT followed by resection benefited from adjuvant chemotherapy (42), but there is no established consensus regarding the benefit and indications of adjuvant therapy in pCR patients. Our analysis showed no significant difference in OS and DFS between patients who received adjuvant therapy and those who did not, indicating that adjuvant therapy might not confer additional survival benefit in patients achieving pCR. Similarly, He et al. observed that adjuvant chemotherapy was unnecessary for patients with locally advanced rectal cancer who achieved pCR through NAT, as it did not improve survival (43). In the real world, the causal relationship between pCR and long-term oncological outcomes is confounded by factors such as neoadjuvant therapy pattern and the biological behavior of the patients. The crucial issue in optimizing treatment strategies for pCR patients is the identification of individuals who are truly cured. The evaluation of minimal residual disease (MRD) through circulating tumor DNA (ctDNA) testing has been preliminarily applied to various solid tumors (44–46). Studies have shown that postoperative ctDNA can reflect the presence of MRD and predict the risk of recurrence in solid tumors, with MRD-positive gastric cancer patients exhibiting a significantly higher risk of recurrence than MRD-negative patients (47, 48). MRD detection can also predict the risk of recurrence in patients undergoing NAT. A phase Ib trial enrolled 32 patients with resectable stage II/III gastroesophageal cancer who underwent neoadjuvant immunotherapy combined with chemoradiotherapy. Patients with undetectable ctDNA at postoperative time points had longer recurrence-free survival (RFS) compared to those with detectable ctDNA (not reached vs. 7.8 months, P = 0.007), with a similar trend observed for OS (49). These findings suggest that ctDNA testing may serve as a valuable tool for assessing MRD status in pCR patients, thereby identifying those who are truly cured and guiding subsequent treatment decisions.

Our study has several limitations. Firstly, it was a single-center study with considerable heterogeneity in treatment regimens. Secondly, although our cohort represented the largest to date, the number of pCR patients receiving neoadjuvant immunotherapy remained limited, and no endpoint events were observed in this subgroup. Further large-scale investigations with extended follow-up are warranted to validate the survival benefits of pCR after neoadjuvant immunotherapy.

## Conclusion

In conclusion, patients with LAGC who achieved pCR exhibited favorable long-term survival outcomes, and adjuvant therapy did not confer additional survival benefits. Although neoadjuvant immunotherapy increased the pCR rate, its impact on the prognosis of pCR patients requires further investigation.

## Data Availability

The raw data supporting the conclusions of this article will be made available by the authors, without undue reservation.
